# Leber Congenital Amaurosis Due to *GUCY2D* Mutations: Longitudinal Analysis of Retinal Structure and Visual Function

**DOI:** 10.3390/ijms22042031

**Published:** 2021-02-18

**Authors:** Samuel G. Jacobson, Artur V. Cideciyan, Alexander Sumaroka, Alejandro J. Roman, Vivian Wu, Malgorzata Swider, Rebecca Sheplock, Arun K. Krishnan, Alexandra V. Garafalo

**Affiliations:** Department of Ophthalmology, Scheie Eye Institute, Perelman School of Medicine, University of Pennsylvania, Philadelphia, PA 19104, USA; cideciya@pennmedicine.upenn.edu (A.V.C.); asumarok@pennmedicine.upenn.edu (A.S.); aroman@pennmedicine.upenn.edu (A.J.R.); vivian.wu@pennmedicine.upenn.edu (V.W.); mswider@pennmedicine.upenn.edu (M.S.); rshepl@pennmedicine.upenn.edu (R.S.); ArunKumar.Krishnan@pennmedicine.upenn.edu (A.K.K.); garafalo@pennmedicine.upenn.edu (A.V.G.)

**Keywords:** cone, clinical trials, rod, optical coherence tomography, outcome measures

## Abstract

Gene augmentation therapy is being planned for *GUCY2D*-associated Leber congenital amaurosis (LCA). To increase our understanding of the natural history of *GUCY2D*-LCA, patients were evaluated twice with an interval of 4 to 7 years between visits using safety and efficacy outcome measures previously determined to be useful for monitoring this disorder. In this group of molecularly-identified LCA patients (*n* = 10; ages 7–37 years at first visit), optical coherence tomography (OCT) was used to measure foveal cone outer nuclear layer (ONL) thickness and rod ONL at a superior retinal locus. Full-field stimulus testing (FST) with chromatic stimuli in dark- and light-adapted states was used to assay rod and cone vision. Changes in OCT and FST over the interval were mostly attributable to inter-visit variability. There were no major negative changes in structure or function across the cohort and over the intervals studied. Variation in severity of disease expression between patients occurs; however, despite difficulties in quantifying structure and function in such seriously visually impaired individuals with nystagmus, the present work supports the use of OCT as a safety outcome and FST as an efficacy outcome in a clinical trial of *GUCY2D*-LCA. A wide age spectrum for therapy was confirmed, and there was relative stability of structure and function during a typical time interval for clinical trials.

## 1. Introduction

Inherited retinal degenerations (IRDs) are a heterogeneous group of disorders that share the feature of abnormal visual function at the level of retinal photoreceptors. The source of photoreceptor dysfunction can be due to a variety of pathophysiological mechanisms, and until a decade ago, IRDs were treated mainly with nutrient supplements aimed to slow the disease [1]. Subsequently, converging information from molecular and retinal biology, animal models, human phenotyping, and therapeutic tools reached a critical mass, and now there is great interest in understanding the underlying mechanisms of IRDs and developing novel treatments [2,3,4,5,6].

Among the more severe IRDs are those clinically classified as Leber congenital amaurosis (LCA), a group of predominantly autosomal recessively inherited diseases with congenital visual impairment, nystagmus (involuntary eye movements), electroretinographic evidence of photoreceptor dysfunction, and a deceptively benign fundus appearance at early stages [7,8]. Some forms of LCA are attractive targets for therapy, not only because of a longstanding unmet need but also because of the potential for improvement of vision from treatment. LCA caused by mutations in the *GUCY2D* gene is theoretically a good candidate for gene augmentation therapy, given the relatively preserved photoreceptor structure despite disproportionately reduced function [9]. The gene *GUCY2D* encodes retinal guanylyl cyclase (retGC1), which modulates phototransduction in rods and cones [10,11].

Retrospective studies of phenotype in *GUCY2D*-LCA have shown dysfunction of cones, rods, or both, and patients have different degrees of abnormality in the central retinal structure [12,13,14]. Considering that this is not a stationary retinopathy, it is important to know whether there is detectable disease progression over a typical time course of a clinical trial (e.g., several years). To date, there have been no prospective natural history studies of the retinal disease in *GUCY2D*-LCA. Valuable observations about change in disease expression have been made by studies reviewing clinical notes and examinations as well as grading available imaging and electroretinography results [14]. To determine outcome measures for future clinical trials of *GUCY2D*-LCA, we previously studied a cohort of 28 molecularly identified patients [13]. We concluded that in this population, a safety outcome (in addition to ocular and systemic examinations) would be segmented and quantified optical coherence tomography (OCT) scans. To define rod- and cone-mediated visual efficacy, we decided that the full-field stimulus test (FST) under dark- and light-adapted conditions with chromatic stimuli would best capture function [13,15].

In the present study, we sought a greater understanding of the natural history of *GUCY2D*-LCA by performing follow-up evaluations (using the OCT and FST outcomes) in a subset of 10 patients from our original cohort. Changes in these parameters between the two visits are quantified and reported here.

## 2. Results and Discussion

### 2.1. Study Population with GUCY2D-LCA

Ten patients with *GUCY2D*-LCA were evaluated on two visits. At the first visit, the patients were part of a larger group to determine outcome measures in *GUCY2D*-LCA in anticipation of clinical trials of gene augmentation therapy [9]. The biallelic mutations in *GUCY2D* have been previously reported [13]. Age at first exam ranged from 7.4 to 37.2 years (median, 13.8 years). The second visit occurred after an interval of 4.0 to 7.3 years (median, 4.6 years) (Supplementary Table S1). All patients had nystagmus and reduced visual acuity (range, 20/80 to light perception).

### 2.2. Serial En Face Near-Infrared Autofluorescence Imaging in GUCY2D-LCA

**Figure 1:**
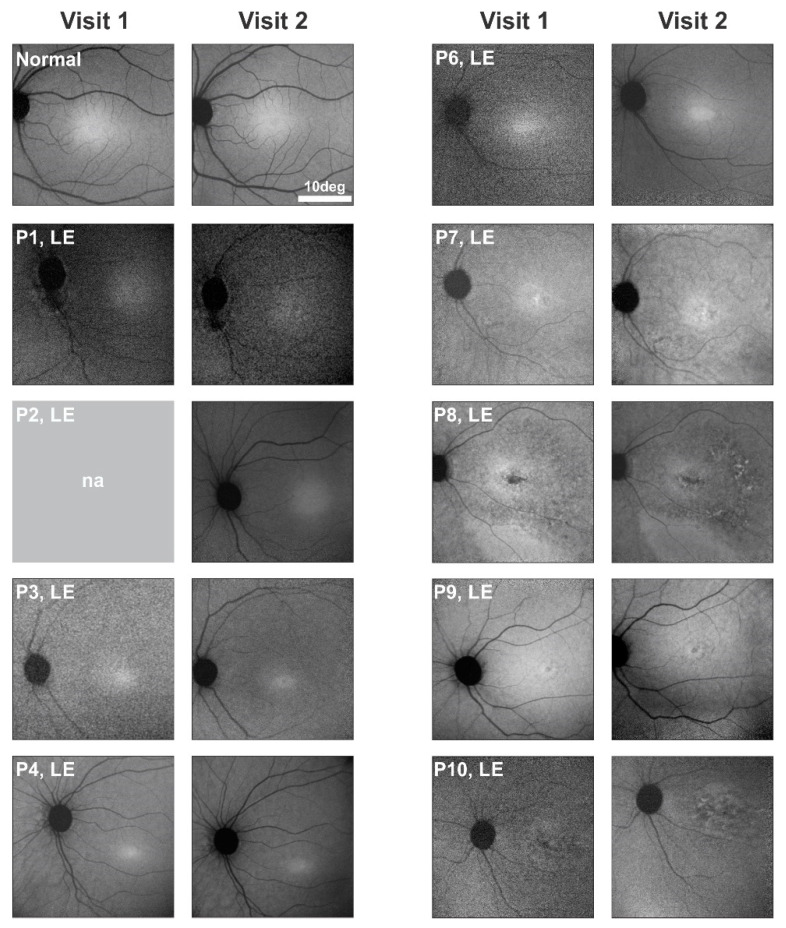
En face near-infrared reduced illuminance autofluorescence (NIR-RAFI) images from the left eye of nine *GUCY2D*-LCA patients at two visits. Also shown are images of a normal eye acquired at ages 7 and 14 years. Images from Visit 1 of P2 and both visits of P5 were not available. The images from P1–P4 and P6 are within normal limits, and there is no obvious change on the second visit. Retinal pigment epithelium (RPE) abnormalities are present in the macula in P7, P8, P9, and P10 at Visit 1; no notable changes in appearance are evident in the images at Visit 2.

Previous reports of ocular phenotype in *GUCY2D*-LCA patients have shown en face fundus images, whether color fundus photographs or fluorescence imaging. There were central retinal abnormalities noted in patients in the fifth decade of life but not at earlier ages; rare examples of peripheral retinopathy have also been reported [10]. We used near-infrared reduced illuminance autofluorescence (NIR-RAFI) imaging [16,17] to examine the central retina of our cohort of patients on two visits to determine if our observations were in keeping with those in other studies that reported en face images in *GUCY2D*-LCA. There were four patients with macular retinal pigment epithelium (RPE) disturbances visible on NIR-RAFI imaging; the youngest patient was P7 at age 19.4, and there were also changes in P8 at age 28.6, P9 at 30.7, and P10 at 37.2 years (Figure 1). The abnormal appearance did not change (by inspection) at the second visit for any of the patients. The results suggest that central retinal RPE abnormalities could be detected in *GUCY2D*-LCA patients about two decades before previous reports; the changes did not appear to advance in size or retinal extent over the interval in this study.

### 2.3. Photoreceptor Nuclear Layer Imaging with OCT as a Quantitative Safety Outcome

**Figure 2:**
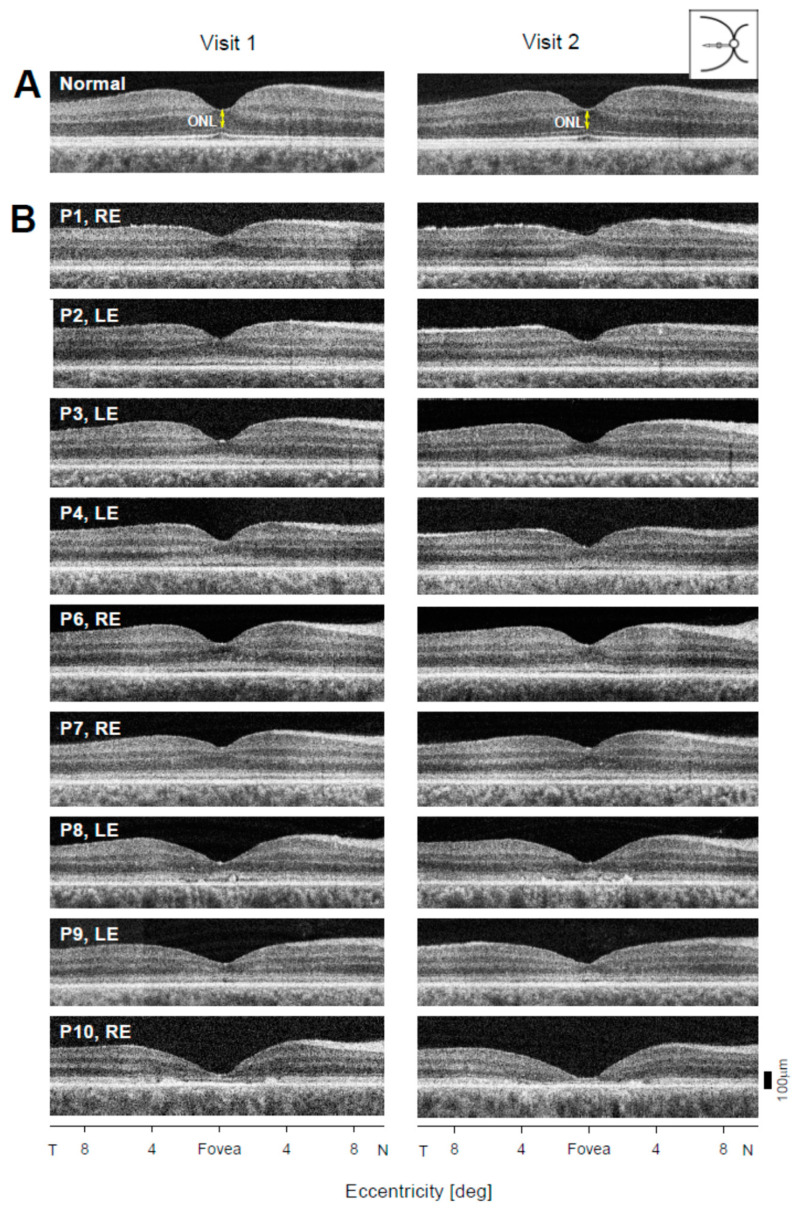
Central retinal optical coherence tomography (OCT) scans of *GUCY2D*–LCA patients at two visits. (**A**) Horizontal scans through the fovea of a normal subject at visits separated by an interval of 9 years. The foveal photoreceptor outer nuclear layer (ONL) is labeled. (**B**) Scans of nine LCA patients (P1–P4, P6–P10) on two visits separated by years, as specified in Supplementary Table S1. T, temporal; N, nasal retina.

**Figure 3:**
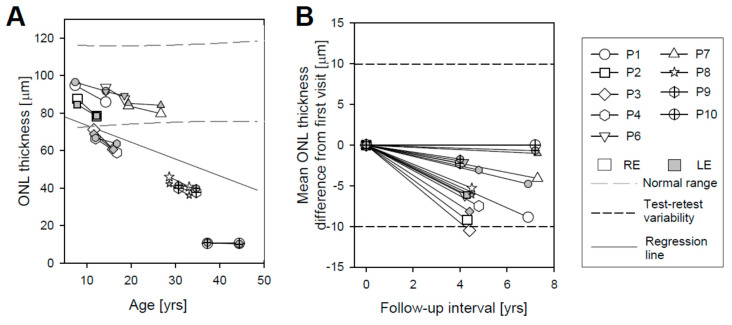
Foveal outer nuclear layer (ONL) measurements in *GUCY2D*–LCA patients at two visits. (**A**) ONL thickness as a function of patient age shows a trend toward reduction of foveal ONL with increasing age. Serial data are connected by solid lines. Black long dashed lines represent the range of normal ONL thickness for comparison [19]. Gray dashed line is the regression line. (**B**) Difference in foveal ONL thickness from first visit versus follow-up interval. Short dashed lines show test–retest variability.

OCT cross-sectional images along the horizontal meridian including the fovea are shown for a 45-year-old individual with normal vision at the two visits separated by 9 years (Figure 2A). Representative horizontal scans through the fovea of one eye for 9 patients on each of the two visits are shown (Figure 2B). The patients are numbered in order of increasing age at first exam (Supplementary Table S1). Inspection of the scans from Visit 1 leads to the impression that there is little difference in foveal ONL thickness in P1–P4, P6, and P7, but P8–P10 appear to have thinner foveal ONL (Figure 2B). P8 also shows apparent deposits in the central few degrees, near the RPE/Bruch’s membrane region; P10 has similar deposits but in the parafoveal area. Quantitation of the foveal ONL thickness at both visits and in both eyes (all except P5) supports the impression from the qualitative observation of the scans (Figure 3A; Supplementary Table S2). P1–P4 and P6–P7 had foveal ONL thicknesses at the first visit that ranged from 66 to 95 µm (median, 85 µm); normal thickness ranges from 73 to 119 µm (ages 8–50 years). Thickness in P8, P9, and P10 measured 46 µm, 40 µm, and 10.6 µm, respectively. Although this is a relatively small sample, the data suggest that foveal ONL thickness in patients in the first and second decades of life can be within or near normal limits, but reduced foveal ONL can occur at later ages. The general tendency of decreasing foveal ONL with age (Figure 3A) was estimated using a linear mixed model with age as a fixed effect and the patient nested with eyes as a random effect. The rate of foveal ONL loss accordingly to this model is 0.90 µm/year (*p* < 0.001).

Are there detectable changes in foveal ONL in the 4–7 year intervals sampled (Figure 3B)? Eight of the nine patients showed relatively small decreases in foveal ONL thickness (P1: RE, 8.9 µm, LE, 4.8 µm; P2: RE, 9.2 µm, LE, 6.1 µm; P3: RE, 10.5 µm, LE, 8.2 µm; P4: RE, 7.5 µm, LE, 3.1 µm; P6: RE 6.1 µm, LE, 2.0 µm; P7: RE, 4.1 µm, LE, 1.0 µm; P8: RE, 5.3 µm, LE, 6.1 µm; P9: RE, 2.2 µm, LE, 1.7 µm); and P10 showed <1 µm changes in both eyes. Changes in average foveal ONL thickness in all but the LE of P3 are within the range of test–retest variability (9.9 µm) which was calculated as 2.77*SD, where SD is the mean within-subjects standard deviation [18]. The observed difference between right and left eyes was also within test–retest variability.

**Figure 4:**
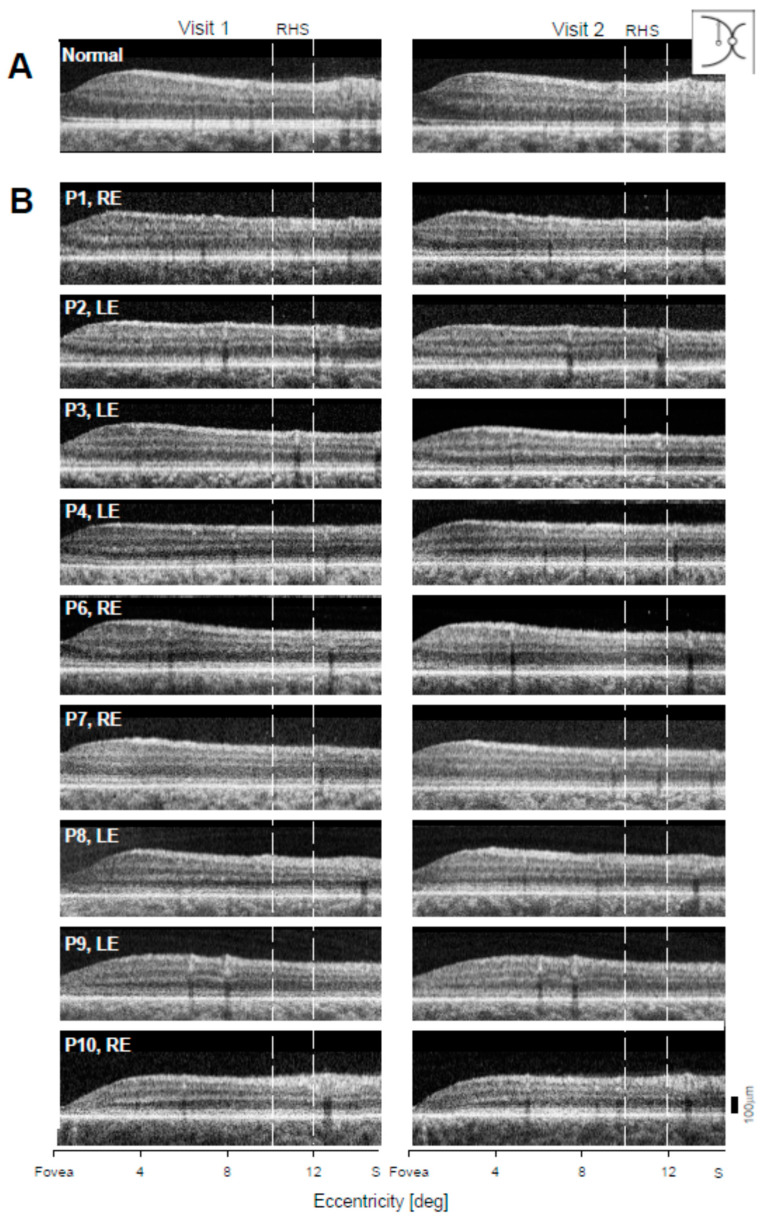
Superior retinal OCT scans in *GUCY2D*–LCA patients at two visits. (**A**) Normal vertical scans in superior direction starting at the fovea. (**B**) Scans of the nine LCA patients (P1–P4, P6–P10) on two visits separated by years, as specified in Supplementary Table S1. S, superior retina. White vertical dashed lines outline the range of eccentricities within the rod hotspot (RHS), where ONL measurements were made.

To measure the rod photoreceptor structure longitudinally in *GUCY2D*-LCA, we quantified ONL in the superior retinal region of highest rod density, known as the rod hotspot (RHS) [20]. Vertical OCT scans beginning at the fovea and crossing the RHS from each visit for the patients are shown (Figure 4). The scans of P5 were not available. The range of eccentricities where ONL measurements were made is marked (white dashed lines). Unlike the fovea ONL thickness, which differed between patients, there was no obvious difference between patients at the RHS.

**Figure 5:**
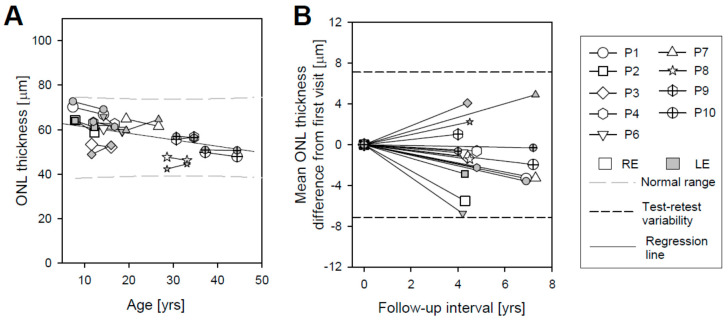
Superior retinal ONL measurements in *GUCY2D*–LCA patients at two visits. (**A**) ONL thickness as a function of patient age. Solid lines connect serial data. Black long dashed lines represent the range of normal ONL thickness for comparison [19]. The gray medium dashed line is the regression line. (**B**) Differences in average ONL thickness from the first visit versus follow-up interval. Short dashed lines show test–retest variability.

Average RHS ONL measurements from individual scans were plotted against age (Figure 5A); the results were also tabulated (Supplementary Table S2). In five patients, ONL thickness demonstrated small decreases in both eyes (P1: RE, 3.3, LE, 3.6 µm; P2: RE, 5.5, LE, 2.9 µm; P4: RE, 0.6, LE, 2.2 µm; P6: RE, 0.8, LE, 6.7 µm; P10: RE, 1.9, LE, 0.3 µm), while P3, P7, and P8 showed a small increase in average ONL thickness for the LE (4.1, 4.9, and 2.2 µm, respectively) and a decrease for the RE (1.2, 3.3, and 1.4 µm, respectively). P9 showed a small increase in average ONL thickness for the RE (1 µm) and a decrease for the LE (0.6 µm). For all nine patients, the change in ONL thickness was within the range of test–retest variability (7.13 µm), which was calculated in the same way as for the foveal region. Test–retest variability is shown (Figure 5B; horizontal short dashed lines). The difference in average RHS ONL thickness between RE and LE for both visits was within test–retest variability for all patients. There appeared to be a weak tendency for decreasing ONL thickness with age in the RHS region, and this was estimated with the same model as foveal ONL (Figure 5A, gray dashed line); the rate of decline was 0.29 µm/year (*p* = 0.01).

### 2.4. Full-Field Stimulus Testing as an Efficacy Outcome in GUCY2D-LCA

Visual sensitivity was measured with the FST system we devised specifically for LCA. FST has been used to quantify vision in many different molecular forms of LCA over the past decade, including as an outcome in our *RPE65*-LCA gene therapy trial [9,13,15,21,22]. FST sensitivities were measured using two color stimuli to determine photoreceptor mediation. Analysis of the sensitivity differences for these chromatic stimuli indicated that all sensitivities were rod-mediated for the dark-adapted (DA) Blue stimulus and cone-mediated for the light-adapted (LA) Red stimuli except in one patient (P6). These stimuli were previously used to define vision in *GUCY2D*-LCA and to compare structure with function [12,13].

**Figure 6:**
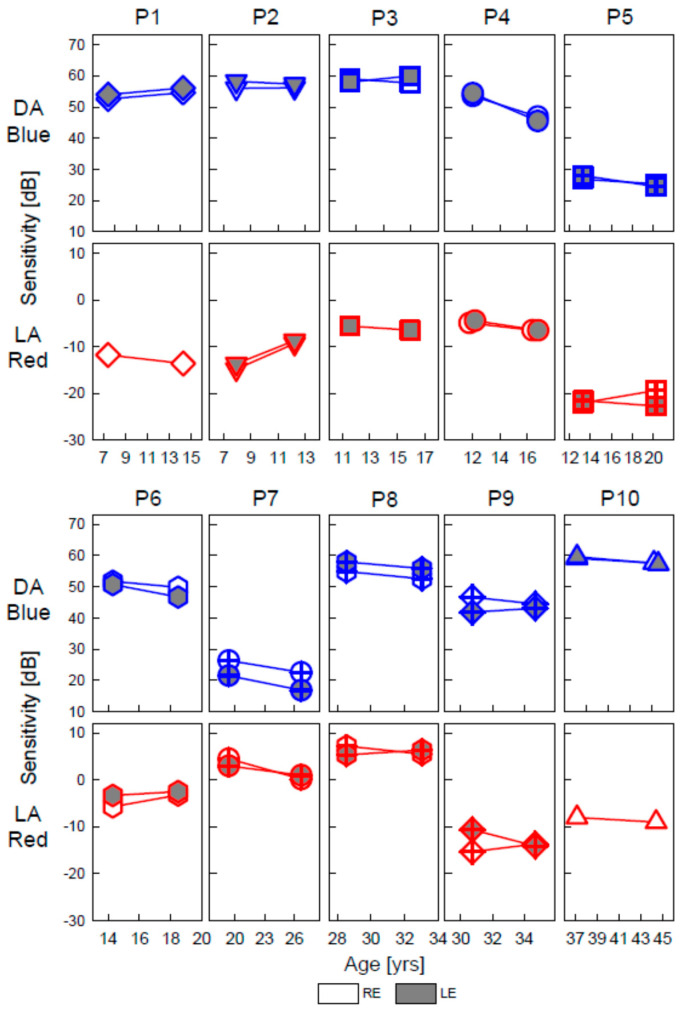
Dark- and light-adapted chromatic full-field stimulus test (FST) results in *GUCY2D*–LCA patients at two visits. Upper panels: Sensitivity for the blue stimulus in the dark-adapted state, which was detected by rods in all cases. Lower panels: Sensitivity for the red stimulus on a 10 phot cd.m^−2^ white background, which was detected by cones in all patients except the RE of P6. Photoreceptor mediation was determined based on chromatic differences: dark-adapted (DA) Blue minus DA Red (not shown); and light-adapted (LA) blue (not shown) minus LA Red. The 0 dB sensitivity level corresponds to −0.57 log_10_ 1/(phot cd.m^−2^).

Sensitivity changes between visits were graphed for each eye of the 10 patients (Figure 6). All numerical changes reported hereafter correspond to the average of both eyes unless specified. For the DA Blue stimulus, P4 had a 7.8 dB decrease. In contrast, P3, who was of similar age and had recordings spanning a comparable interval, had only a 0.5 dB decrease. P1, representing a younger age group and a slightly longer interval between visits, had a 2.2 dB increase in sensitivity to this stimulus. P10, over a similar interval but in the fourth decade of life at the first visit and fifth decade upon return, showed a 1.8 dB decrease in sensitivity. The other six patients, P2, P6, P7, P5, P9, and P8, showed decreases of 0.4, 3.1, 4.2, 2.4, 0.42, and 2.2 dB over intervals of 4.3, 4.2, 7.3, 6.9, 4.0, and 4.5 years, respectively.

**Figure 7:**
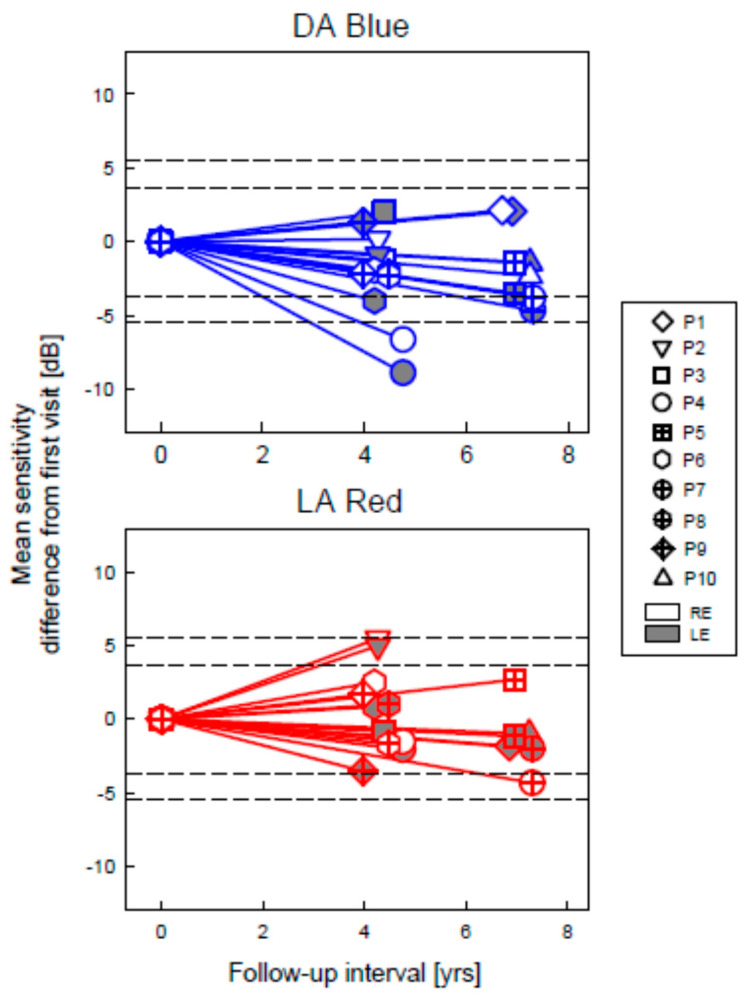
FST sensitivity changes from first visit in GUCY2D–LCA patients as a function of follow-up interval. Symbols, testing conditions, and stimulus levels correspond to those in Figure 6. Dashed lines indicate 95 and 99% confidence intervals for test–retest variability.

For the DA Blue stimulus, what should be considered a statistically significant change? The changes in mean sensitivity versus follow-up time in the patients compared with our published FST inter-visit variability estimate with 95% and 99% confidence limits [15] are shown (Figure 7). The sensitivity change across the follow-up interval did not exceed the 95% limits in seven of the ten patients. Decreases in P4 and P7 (both eyes) and P6 (left eye only) did reach the limit.

For the LA Red stimulus, all patients showed changes in sensitivity between the visits, but most did not exceed the inter-visit variability limits (Figure 7). Average decreases in sensitivity were 1.8, 0.84, 1.8, 1.0, 3.2, 0.7, and 0.6 dB over follow-up intervals of 4.8, 4.4, 6.9, 7.2, 7.3, 4, and 4.5 years for P4, P3, P1, P10, P7, P9, and P8, respectively. Average increases in sensitivity were 5.2, 1.7, and 0.7 dB over follow-up intervals of 4.3, 4.2, and 6.9-years for P2, P6 and P5, respectively. The decrease in the right eye of P7 and the increase in both eyes of P2 exceeded the 95% inter-visit variability limits. The increase in P2 may reflect the maturation of the patient over this interval and an ability to perform the test more reliably at the second visit.

An overall analysis (combining all data from all eyes) for sensitivity change versus time after baseline was performed separately for LA Red and DA Blue stimuli. In the LA Red results, the effect of time was not significant (*p* = 0.26). In the DA Blue results, there was a small decreasing trend of 0.28 log units per decade (*p* ≤ 0.001). This tendency resembles data found in another form of LCA with seriously impaired vision [23].

## 3. Summary and Conclusions

Patients with *GUCY2D*-LCA were assessed twice using structural and functional examinations with an interval of 4 to 7 years between visits. Conclusions about disease progression are as follows:

(1) En face imaging performed with NIR-RAFI methods revealed no abnormalities in the first two decades of life, but there were central RPE changes in the older patients of this cohort. Progression of these changes on the second visit was not evident.

(2) Cross-sectional retinal imaging at the fovea and in the superior retina was performed with OCT. Changes were measurable, given carefully-acquired high quality scans (despite the nystagmus), in 9 of 10 patients. At the fovea, only one patient in one eye had a decrease in thickness that would be considered outside the range of inter-visit variability. Changes in the superior retinal ONL were all attributable to inter-visit variability and not to structural changes.

(3) Visual sensitivity was quantified separately for rods and cones using dark- and light-adapted chromatic FST. Analyzed individually, most patients did not show significant changes in rod function. Exceptions were the decreases in rod function in both eyes of two patients (1.0 and 0.4 log unit over 4.8 and 7.3 years) and in one eye of a third patient (0.3 log unit over 4.2 years). One of these patients also showed a decrease of similar magnitude in cone function, but only in one eye (0.4 log unit in 7.3 years). There was a fourth patient showing an increase in cone function over a 4.3-year interval. A trend analysis combining all available data showed a small decrease in rod function (0.28 l.u. per decade for DA Blue), and a rate for LA Red that was not statistically significant.

In summary, given the appropriate expertise in recording, analyzing, and interpreting relevant data from these seriously visually impaired *GUCY2D*-LCA patients, change could be detected with the quantitative methods used. The detected changes in this group of patients, however, were mostly attributable to inter-visit variability. There were no major negative changes in structure and/or function in this cohort and over the intervals studied.

## 4. Materials and Methods

### 4.1. Subjects

Ten patients with *GUCY2D*-LCA were evaluated at two visits. At the first visit, the patients ranged in age from 7 to 37 years, and they were re-evaluated after intervals of approximately 4–7 years. All patients had molecular testing in a CLIA (Clinical Laboratory Improvement Amendments)-approved laboratory. Procedures followed the Declaration of Helsinki, and the study was approved by the relevant institutional review board. Informed consent, assent, and parental permission were obtained, and the work was HIPAA (Health Insurance Portability and Accountability Act)-compliant.

### 4.2. Near-Infrared Autofluorescence

A confocal scanning laser ophthalmoscope (Spectralis HRA; Heidelberg Engineering, Heidelberg, Germany) was used to record en face images and estimate RPE health. NIR-RAFI was performed using methods previously described [16,17]. For NIR-RAFI, excitation at 790 nm was used at 100% laser power setting and 105% detector sensitivity. All images were acquired at high speed mode (30° × 30° square field sampled onto 768 × 768 pixels) and with the automatic normalization feature turned off. The manufacturer’s ART (Automatic Real Time-function) feature was used whenever possible with a 21-frame average.

### 4.3. Optical Coherence Tomography

Cross-sectional images of the retina were obtained with a spectral-domain OCT instrument (RTVue-100; Optovue Inc., Fremont, CA, USA). The scans were 30° in length along the horizontal and vertical meridians to study the retinal changes at the fovea and at the rod-rich region 10–15° in the superior retina (rod hotspot, RHS). To measure ONL thickness at the fovea, three horizontal scans from each visit for each patient were selected based on the shape of the foveal depression (criteria for selection: similar contour of the foveal depression in all three scans, deepest pit, no inner retinal layers or thinnest ones, and the same position of blood vessels). The foveal ONL thickness was defined as the distance between the internal limiting membrane and outer limiting membrane, and it was measured with custom programs (MATLAB R2018a, MathWork, Natick, MA, USA). Unlike at the fovea, where one measurement was performed, ONL thickness at RHS represents the average of five measurements along the vertical scan at 0.5-degree increments within a range of 10–12° superiorly. These measurements were also performed on three scans except for P1, LE, and P10, LE, where only two scans were available.

### 4.4. Visual Function

FST was performed to estimate perceptual light sensitivity as previously described [11,17]. Stimuli were 200 ms photopically-matched blue and red flashes (peak 465 nm and 637 nm, respectively) in the dark-adapted (DA) and light-adapted (LA, steady white 10 phot cd.m^−2^ background) states [15,21] using a ganzfeld stimulator (Colordome; Diagnosys LLC, Littleton, MA, USA). Participants responded when they perceived the lights presented. Several threshold estimates for each color were obtained within a session and averaged. Photoreceptor mediation was determined by sensitivity differences between the chromatic stimuli. Differences higher than 3.2 dB indicate rod-mediation of the blue stimuli, and those lower than 22.2 dB indicate cone mediation of the red stimuli.

## Supplementary Materials

Supplementary materials are available online at https://www.mdpi.com/1422-0067/22/4/2031/s1.
