# Supplementary material for: Leber Congenital Amaurosis Due to GUCY2D Mutations: Longitudinal Analysis of Retinal Structure and Visual Function
Source: Int J Mol Sci. 2021 Feb 18;22(4):2031. doi: 10.3390/ijms22042031 (PMC7922686; doi:10.3390/ijms22042031)
Supplement: Supplementary file 1 [file ijms-22-02031-s001.pdf]

International Journal of
Molecular Sciences

MDPI

Supplementary Material

**Leber Congenital Amaurosis Due to *GUCY2D* Mutations:
Longitudinal Analysis of Retinal Structure and Visual Function**

Table S1: *GUCY2D*-LCA patients and visit intervals

Patient Number	Gender	Age at Visit 1	Age at Visit 2	Follow-up Duration (years)	Patient Number in Previous Report [9]
P1	M	7.4	14.3	6.9	P3
P2	F	7.9	12.2	4.3	P5
P3	F	11.6	16.0	4.4	P11
P4	M	12.0	16.8	4.8	P12
P5	F	13.3	20.2	6.9	P14
P6	F	14.3	18.5	4.2	P16
P7	F	19.4	26.7	7.3	P18
P8	F	28.6	33.1	4.5	P22
P9	M	30.7	34.7	4.0	P23
P10	F	37.2	44.4	7.2	P25

Supplementary Material

Leber Congenital Amaurosis Due to *GUCY2D* Mutations:
Longitudinal Analysis of Retinal Structure and Visual Function

Table S2: Outer nuclear layer thickness in *GUCY2D*-LCA patients on two visits

Patient Number	Age at visit (years)	Foveal ONL (µm)		RHS ONL (µm)	
		OD	OS	OD	OS
P1	7.4	94.7	96.4	70.2	72.7
	14.3	85.9	91.7	66.9	69.1
P2	7.9	87.6	84.5	64.2	64.0
	12.2	78.4	78.4	58.7	61.1
P3	11.6	71.2	68.8	53.4	48.9
	16.0	60.7	60.7	52.1	53.0
P4	12.0	66.5	66.8	63.2	63.6
	16.8	59.0	63.7	62.6	61.3
P6	14.3	93.7	91.3	60.5	65.8
	18.5	87.6	89.3	59.7	59.1
P7	19.4	83.8	85.2	64.8	59.7
	26.7	79.7	84.2	61.5	64.6
P8	28.6	46.0	42.3	47.6	42.3
	33.1	40.7	36.1	46.2	44.6
P9	30.7	40.0	41.6	55.6	57.0
	34.7	37.8	39.9	56.6	56.4
P10	37.2	10.6	10.9	49.8	50.9
	44.4	10.6	10.2	47.8	50.7

ONL, outer nuclear layer; RHS, rod hotspot
